# Distinct spatiotemporal subtypes of amyloid deposition are associated with diverging disease profiles in cognitively normal and mild cognitive impairment individuals

**DOI:** 10.1038/s41398-023-02328-2

**Published:** 2023-02-02

**Authors:** Yuqing Sun, Yuxin Zhao, Ke Hu, Meng Wang, Yong Liu, Bing Liu, for the Alzheimer’s Disease Neuroimaging Initiative

**Affiliations:** 1 State Key Laboratory of Cognitive Neuroscience and Learning, Beijing Normal University, Beijing, 100875 China; 2 School of Artificial Intelligence, University of Chinese Academy of Sciences, Beijing, 100049 China; 3 Brainnetome Center and National Laboratory of Pattern Recognition, Institute of Automation, Chinese Academy of Sciences, Beijing, 100190 China; 4 School of Artificial Intelligence, Beijing University of Posts and Telecommunications, Beijing, 100876 China; 5 Chinese Institute for Brain Research, Beijing, 102206 China

**Keywords:** Neuroscience, Predictive markers

## Abstract

We aimed to investigate the relationship between spatiotemporal changes of amyloid deposition and Alzheimer’s disease (AD) profiles in cognitively normal (CN) and those with mild cognitive impairment (MCI). Using a data-driven method and amyloid-PET data, we identified and validated two subtypes in two independent datasets (discovery dataset: *N* = 548, age = 72.4 ± 6.78, 49% female; validation dataset: *N* = 348, age = 74.9 ± 8.16, 47% female) from the Alzheimer’s Disease Neuroimaging Initiative across a range of individuals who were CN or had MCI. The two subtypes showed distinct regional progression patterns and presented distinct genetic, clinical and biomarker characteristics. The cortex-priority subtype was more likely to show typical clinical syndromes of symptomatic AD and vice versa. Furthermore, the regional progression patterns were associated with clinical and biomarker profiles. In sum, our findings suggest that the spatiotemporal variants of amyloid depositions are in close association with disease trajectories; these findings may provide insight into the disease monitoring and enrollment of therapeutic trials in AD.

## Introduction

Alzheimer’s disease (AD) is characterized by the accumulation of extracellular beta-amyloid (Aβ) proteins, which affects subsequent events that include tau deposition, synaptic and neuronal loss, and cognitive decline [1, 2]. Currently, amyloid pathology has become an important diagnostic criterion for AD, promoting the use of cerebrospinal fluid (CSF) and positron emission tomography (PET) measurements as equivalent measures [3–5]. Numerous studies have detected increased levels of amyloid accumulation in PET in individuals who were either cognitively normal (CN) or had mild cognitive impairment (MCI) [6, 7]. However, these individuals have shown remarkable variability in clinical performance. Therefore, further exploration of amyloid accumulation, such as early detection and stratification and linking it with downstream events, is required to aid the clinical trials.

Amyloid accumulation affects different brain regions at different time points, producing abundant spatiotemporal variations. Characterizing and staging the regional amyloid deposition spread could enable the earlier detection and stratification of amyloid accumulation. Furthermore, such regional progression patterns have been found to be associated with cognitive impairment [6, 8–11]. Post-mortem neuropathology previously identified a 4-stage model of amyloid deposition, which begins in the associative neocortex, then affects limbic and primary sensory-motor areas, and finally impacts subcortical structures, forming the basis for Braak staging [8, 9]. Amyloid PET studies also explored and verified Braak staging in vivo using discrimination or data-driven methods [6, 10, 12]. These studies assumed that all individuals follow a common progression pattern, and the inherent assumption has limited the discovery of subtypes. Recently, spatiotemporal variations of neuropathology in AD spectrum have been decoded properly by identifying distinct subtypes with distinct regional progression patterns [13, 14]. It is now possible to explore the relationship between spatiotemporal changes of amyloid deposition and molecular and cognitive biomarkers early on, which may lead to more differentiated population stratification and is crucial for improving the early prediction of AD pathophysiology [15].

In this study, we hypothesized that heterogeneous disease characteristic is associated with amyloid spatiotemporal accumulation in CN and MCI. To directly test this hypothesis, we studied multiple regional progression patterns on PET imaging to decode the spatiotemporal variations of amyloid deposition using the Subtype and Stage Inference (SuStaIn) model [16] in CN and MCI. SuStaIn identifies subtypes with common disease progression patterns by combining clustering and disease progression modeling using only cross-sectional datasets, which contain snapshots of biomarker measurements. Furthermore, the subtype and temporal stage of each subject can be inferred probabilistically based on the model [16]. Using SuStaIn with Alzheimer’s Disease Neuroimaging Initiative (ADNI) data, we found two subtypes in both the discovery and validation datasets, which included a broad range of individuals who were either cognitively normal (CN) or had mild cognitive impairment (MCI). We compared the two subtypes and assessed their stages with respect to clinical, genetic, and biomarker characteristics that are known to be associated with AD [i.e., Mini-Mental State Examination (MMSE), memory, executive function, language function, visuospatial function, apolipoprotein E ε4 allele (*APOE* ε4), CSF Aβ, total tau (t-tau), and phosphorylated tau (p-tau)]. Moreover, we validated our results in the validation dataset.

## Material and methods

### Participants

The data used in this study was acquired from the ADNI, and the up-to-date information is accessible on the website (www.adni-info.org). The ADNI study was approved by all the Institutional Ethical Review Boards of all the participating centers, and all the participants provided written informed consent to participate in the study. The data inclusion criteria used in this study were as follows. MCI criteria were (1) MMSE score between 24 and 30 (inclusive); (2) global Clinical Dementia Rating (CDR) of 0.5. CN was (1) MMSE score between 24 and 30 (inclusive); (2) global CDR of 0. A set of 896 participants with available amyloid PET and T1-weighted MRI was used in the analysis. The data were split into discovery datasets (ADNI 2, *N* = 548) and validation datasets (ADNI 1/GO/3, *N* = 348). Table 1 presents the characteristics of the above study data. Several profiles were only available for a subset of individuals across the two datasets, including CSF biomarkers (71.3%) and *APOE* ε4 allele carriage (91.2%). CSF samples were acquired through lumbar puncture. Data were retrieved from the ADNIMERGE.csv. Composite scores for memory, executive, language, and visuospatial functions were available in the UWNPSYCHSUM file from ADNI repository [17, 18]. Longitudinal clinical follow-up was available for a subset of individuals (95.3 and 72.1% in the discovery and validation datasets, respectively). The conversion event was determined by whether individuals converted to dementia within 6 years.

**Table 1:** Sample characteristics.

	ADNI2	ADNI1/GO/3	*P* value
Demographics			
*N* (CN/MCI)	247/301	159/189	-
Age	72.4 ± 6.77	74.9 ± 8.14	<0.0001
Gender (M/F)	280/268	184/164	0.5377
Education	16.5 ± 2.59	16.1 ± 2.8	0.0152
Clinical domains			
MMSE	28.5 ± 1.62	28.5 ± 1.57	0.4258
Memory	0.6 ± 0.76	0.7 ± 0.69	0.1539
Executive function	0.6 ± 0.92	0.7 ± 0.85	0.0644
Language function	0.6 ± 0.79	0.6 ± 0.78	0.2362
Visuospatial function	0.1 ± 0.70	0.2 ± 0.66	0.0250
^a^Conversion (CN/MCI)	4/83	3/36	-
CSF biomarkers			
*N* (CN/MCI)	210/276	27/126	-
Aβ	1092.4 ± 438.89	1090.9 ± 471.62	0.1507
t-tau	265.3 ± 122.29	268.7 ± 105.23	0.1316
p-tau	25.1 ± 13.4	25.6 ± 12.25	0.2005
Genotype			
*APOE* ε4 (0/1/2)	319/188/41	183/78/8	0.0057
Amyloid-PET positive ratio			
Cutoff of 1.1	48.1%	42.2%	-

Average values are reported as mean ± SD. *N* = sample size, *MCI* mild cognitive impairment, *CN* cognitively normal, *F* female, *M* male, *MMSE* Mini-Mental State Examination, *CSF* cerebrospinal fluid, *Aβ* beta-amyloid, *t-tau* total tau, *p-tau* phosphorylated tau.

^a^Persons who converted to dementia within 6 years follow-up. *APOE* ε4 genotype and CSF biomarkers were not available for all participants. Amyloid-PET positive ratio is the proportion of individuals with global PET measures greater than 1.1.

### Image data acquisition and preprocessing

Structural MRI data were acquired on 3T MRI scanners in ADNI2, ADNIGO, and ADNI3 or on 1.5T MRI scanners in ADNI1. Amyloid PET images were obtained using ^18^F-florbetapir radiotracers. The detailed parameters of the imaging data can be found online (http://adni.loni.usc.edu). To increase the uniformity in the multicentric data, all PET scans underwent standardized pre-processing steps in ADNI. Briefly, six five-minute frames or four five-minute frames were acquired 30 to 60 min post-injection. To reduce the effects of head motion, each extracted frame was co-registered to the first frame (acquired at 30–35 min). The first frame and co-registered frames were recombined into a co-registered dynamic image set. Frames in the image set were averaged and reoriented into standard space (voxel grid size 160 × 160 × 96, voxel size 1.5 mm cubic). Spatial re-orientation and intensity normalization had been applied to scans in the image set. Finally, the images were smoothed to the lowest resolution scanners (8 mm full-width at half-maximum uniform isotropic). To obtain a standard uptake value ratio (SUVR) to form a voxel-wise map, the preprocessed amyloid PET scans were proportionately scaled by the mean uptake values for the whole cerebellum, which is most commonly used for scaling Florbetapir-PET data [19, 20].

Each amyloid PET scan was rigidly co-registered to the time-matched T1-weighted MRI. Then, all the PET scans were spatially normalized into MNI standard space using the registration parameters derived from the T1-weighted MRI normalization. All the imaging data were processed by applying the cubic spline interpolation scheme implemented in the Aladin algorithm [21]. Finally, we computed the average SUVRs across the 11 regions of interest (ROIs), which were the bilateral regions of the frontal, temporal, parietal, and occipital lobes as well as of the insula, amygdala, cingulate cortex, hippocampus, thalamus, basal ganglia, and cerebellum, all of which are in the first-level of the Human Brainnetome atlas [22]. Each ROI SUVR was adjusted by demographic data (age, gender, and education) using the linear regression model in the ADNI2 and ADNI1/GO/3 datasets.

### Uncovering the spatiotemporal subtypes based on SuStaIn model

Based on the above ROI SUVRs for each individual, we used the SuStaIn model [16] to identify plausible spatiotemporal subtypes of amyloid accumulation. SuStaIn is a probabilistic data-driven method developed to uncover the spatiotemporal variance of biomarkers. This algorithm, the detailed formalization of which has been described previously [16], was implemented by the group from the UCL’s Progression of Neurological Disease and is available on the website (https://github.com/ucl-pond). SuStaIn estimates the maximum likelihood sequence of biomarker transitions to abnormalities within each of the data-driven subgroups; this allows the reconstruction of subtypes with a range of disease stages [16]. Moreover, SuStaIn can calculate the probability that any individual falls into a specific stage of each subtype. Individuals who have normal biomarker measurements in all regions are classified as stage0 and are not assigned to any subtype.

Based on biomarker measurements within different ROIs, the disease progression model is represented by a set of optimal sequences, which are modeled as a succession of severity *z*-scores for each biomarker and represent the distinct subtypes [16]. In this case, we calculated regional amyloid *z*-scores by normalizing the mean and the standard deviation of the cognitively normal subjects. We defined 11 variables with one *z*-score so that each subtype included 11 stages, which ranged from one (the earliest stage) to 11 (the last stage). Previous studies used consistent severity *z*-scores for all biomarkers [13, 16], but differential cutoffs need to be used due to noise variations between brain regions [23, 24]. Here, we used the amyloid positive ratio on the global signal as the regional positive ratio to determine specific regional cutoffs, and we selected the most common cutoff of 1.1 [20, 25]. Then, the ratio was used as the regional Aβ-abnormality ratio (percentage of individuals showing suprathreshold z-score) of 11 ROIs. Based on the z-scores distribution of each ROI and the regional Aβ-abnormality ratio, we determined region-specific z-score cutoffs of 11 ROIs. We trained the SuStaIn model in the discovery dataset (amyloid-positivity ratio at 48.1%, Table 1) to identify the subtypes. We fitted the model using up to 4 subtypes and used ten-fold cross-validation to choose the optimal number based on Cross-Validation Information Criterion (CVIC) [16].

### Replicability and stability of subtypes

To evaluate the consistency between subtypes, we measured the similarities of subtype progression patterns by evaluating the Kendall rank correlation. The Kendall rank correlation ranges from −1 (inverse sequences) to 1 (identical sequences), and an expectation of 0 means independent sequences. To assess the replicability of subtypes, we reran the SuStaIn model on the independent validation dataset using the same steps. We then compared the subtype progression patterns identified in the discovery and validation datasets to examine whether we could identify the same subtypes. To test the stability of each subtype, we compared the consistency of the sequences across cross-validation folds. The averaged results of subtypes in each dataset were given. In addition, we took 1,000,000 Markov Chain Monte Carlo (MCMC) samples to evaluate the uncertainty of each sequence, a process which has been used in many previous studies [13, 16]. Furthermore, SuStaIn enables the probabilistic assignment of individuals to the most probable subtype and stage. Meanwhile, the distribution of individuals across subtypes and stages was obtained.

### Examination of subtype characteristics

To examine the subtype-specific profiles, we compared individuals of different subtypes with respect to demographic (age, gender, and education), clinical (MMSE, memory, executive, language, and visuospatial function), genetic (*APOE* ε4), and biomarker characteristics (CSF Aβ, t-tau, and p-tau) by ANOVA or chi-square test, depending on which was appropriate. These analyses were false discovery rate (FDR)-corrected for multiple comparisons. To evaluate the progression of each subtype, we assessed the relationship between the stages of each subtype and these characteristics using Spearman correlation. To test the usefulness of each subtype to predict conversion to dementia, we used Cox proportional hazards models with subtypes or stages as predictors, adjusted for age, gender, and education and took censoring into account with a maximum follow-up of 6 years. The survival findings are illustrated graphically, as discussed later in the paper. In all the analyses, the covariates (age, gender, and education) were adjusted, and the continuous variables were centered and scaled.

## Results

### Amyloid spatiotemporal subtypes in CN and MCI

**Fig. 1:**
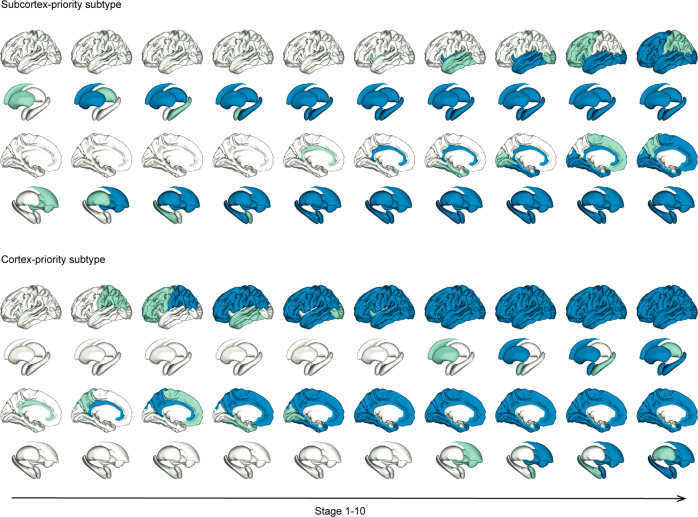
Regional progression patterns of amyloid accumulation identified via the discovery dataset (ADNI2). Green shows the abnormal regions in the numbered stage and blue indicates abnormal regions from the previous stage. The last stage, cerebellum, is not shown here. In the subcortex-priority subtype, amyloid abnormality begins in several subcortex regions and successively affects the cingulate, amygdala, and insula, and finally the cortical regions. In the cortex-priority subtype, the cingulate is the first abnormal region, followed by cortical areas, and then the insula and subcortical regions.

We applied the SuStaIn model to cross-sectional amyloid-PET data to explore distinct subtypes that could be characterized by regional progression patterns. In ADNI2, we evaluated the CVIC of the cluster solutions by cross-validation and found two optimal and converged subtypes. The two progression patterns started with different regions and finally progressed to the cerebellum via different pathways. Figure 1 shows the two distinct progression patterns without the last stage (cerebellum). In subtype 1, the subcortical regions, followed by the cingulate and then the insula became abnormal earlier than the cortical areas. We refer to this trajectory as the subcortex-priority subtype. In subtype 2, the cingulate was the first abnormal region followed by the cortical regions, then the insula, and from there the subcortical regions, so we termed it as the cortex-priority subtype.

### Reproducibility of the subtypes between the independent datasets

**Fig. 2:**
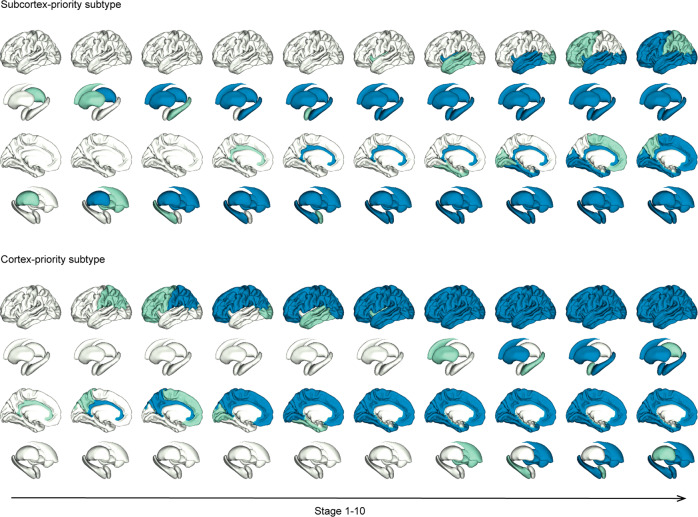
Amyloid abnormality patterns reproduced in the validation dataset (ADNI1/GO/3). Green represents the abnormal regions in the numbered stage and blue indicates abnormal regions of the previous stage. The last stage, cerebellum, is not shown here. The progression pattern of amyloid abnormality in the subcortex-priority subtype evolves from the subcortical regions, over the cingulate and insula, to the cortical regions. The progression pattern of amyloid abnormality in the cortex-priority subtype evolves from the cingulate, to the cortical regions, to the insula, and finally to the subcortical regions.

We reran the SuStaIn model on the validation dataset with the cutoffs in the discovery dataset. The model identified two optimal subtypes, again a cortex-priority subtype and a subcortex-priority subtype (Fig. 2). We compared the progression patterns of the identical subtypes across the two datasets and found an average consistency of 85.5%. In the subcortex-priority subtype of the validation dataset, abnormalities of the thalamus and basal ganglia appeared in the very first stages, followed by a similar order to that identified in the subcortex-priority subtype of the discovery dataset, but the specific order of the amygdala and cingulate were swapped. In the cortex-priority subtype, the cingulate was the first region to show abnormality followed by the cortical regions, and the subcortical regions became abnormal at the end. Unlike the previous cortex-priority subtype, the occipital cortex became abnormal earlier than the temporal, and the hippocampus changed before the amygdala. In general, however, the cortex-priority and subcortex-priority subtypes were replicated in the independent datasets.

### Stability of the two subtypes

The stability of each subtype was tested across cross-validation folds. We found that the subtypes were robust in both datasets, giving an average consistency of 98.0% and 98.8%, separately. Central progression patterns and their variances were further estimated by MCMC. Positional variance diagrams showed good positional stability (Supplementary Fig. 1). These findings indicated the stability of the subtypes. Furthermore, individuals were probabilistically assigned to subtypes and stages (Supplementary Figs. 2 and 3), which were identified based on the cut-offs and the two independent datasets, separately. Fewer than 4% of the individuals were classified to stage0 or the last stage (cerebellum in all subtypes) across the datasets. These individuals could not be assigned to a subtype and were excluded from further analysis. The progression patterns we identified not only represented amyloid deposition over time but also provided reliable utility for individual stratification.

### Demographics, genetic and biomarker characterizations of the two subtypes

In both datasets, there were no differences in age, gender, and education between the cortex-priority and subcortex-priority subtypes (all *P*s > 0.05). We further evaluated the *APOE* ε4 frequency between the subtypes (Table 2). In ADNI2, the proportion of *APOE* ε4 carriers were lower in the subcortex-priority subtype than in the cortex-priority subtype (χ^2^(2) = 38.07; *P*_FDR_ < 0.0001). We validated the result in ADNI1/GO/3 (χ^2^(2) = 14.84; *P*_FDR_ = 0.0015), which further highlighted the significant difference in *APOE* ε4 distributions between the two subtypes.

**Table 2:** Clinical, CSF biomarker, and genetic comparisons between subtypes.

	ADNI2				ADNI1/GO/3			
	Subcortex-priority (*n* = 257)	Cortex-priority (*n* = 274)	*F* value	*P*_FDR_ value	Subcortex-priority (*n* = 217)	Cortex-priority (*n* = 122)	*F* value	*P*_FDR_ value
Clinical profiles								
MMSE	28.8 ± 1.36	28.2 ± 1.76	21.57	<0.0001	28.6 ± 1.51	28.2 ± 1.65	5.64	0.0232
Memory	0.9 ± 0.68	0.4 ± 0.79	47.00	<0.0001	0.8 ± 0.68	0.6 ± 0.68	6.41	0.0177
Executive function	0.8 ± 0.90	0.4 ± 0.91	27.07	<0.0001	0.8 ± 0.85	0.5 ± 0.78	10.55	0.0029
Language function	0.7 ± 0.70	0.4 ± 0.84	23.77	<0.0001	0.6 ± 0.80	0.6 ± 0.69	0.44	0.5732
Visuospatial function	0.1 ± 0.68	0.1 ± 0.72	0.19	0.6569	0.2 ± 0.67	0.2 ± 0.65	0.01	0.9685
CSF Biomarkers								
Aβ	1346.1 ± 376.26	861.4 ± 359.82	212.36	<0.0001	1319.1 ± 424.25	764.8 ± 333.06	75.11	<0.0001
t-tau	242.8 ± 104.91	290.0 ± 133.92	18.19	<0.0001	246.6 ± 94.64	299.1 ± 111.81	10.18	0.0030
p-tau	22.0 ± 10.73	28.5 ± 14.96	28.79	<0.0001	22.3 ± 10.39	30.3 ± 13.27	17.95	0.0004
Genotype								
*APOE* ε4 (0/1/2)	182/67/8	125/117/32	38.07^a^	<0.0001	159/52/5	66/47/9	14.84^a^	0.0015

Data are presented as *n* or mean ± SD. Tests are based on chi-square and ANOVA when appropriate.

^a^means χ^2^. *MMSE* Mini-Mental State Examination, *CSF* cerebrospinal fluid, *Aβ* beta-amyloid, *t-tau* total tau, *p-tau* phosphorylated tau.

**Fig. 3:**
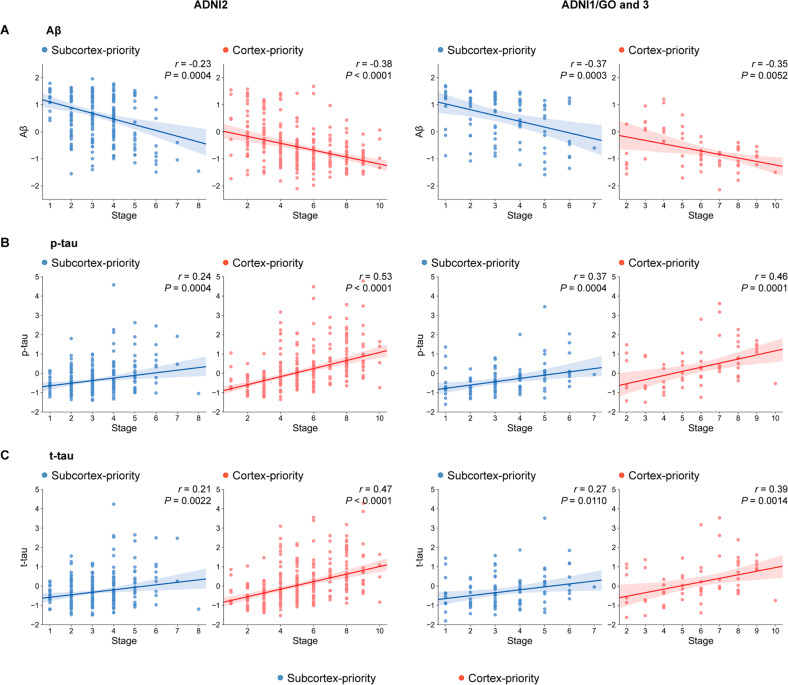
Biomarkers profiles are correlated with progression patterns of amyloid in each subtype. **A** Higher progression stages of the subcortex-priority and cortex-priority subtypes were associated with lower CSF Aβ across the discovery and validation datasets. **B** Higher stages of each subtype were associated with increased levels of CSF p-tau across the two datasets. **C** Similarly, the significant associations between stages of each subtype and CSF t-tau. All associations were tested by Spearman correlation while controlling covariates (age, gender and education).

Table 2 summarizes the difference in the CSF biomarkers (Aβ, t-tau, and p-tau) between the subtypes. In comparison with the subcortex-priority subtype, subjects in the cortex-priority subtype had a lower level of Aβ and higher concentration of t-tau and p-tau when controlling for the covariates. This means that the cortex-priority subtype had more severe AD profiles. In both the subcortex-priority subtype and the cortex-priority subtype, the stages correlated positively with Aβ, as expected (Fig. 3). Furthermore, individuals showed increasing t-tau and p-tau concentrations over the follow-up stages. These findings were consistent between the two datasets. In general, we found that the cortex-priority and subcortex-priority subtypes showed distinct genetic and biomarker characteristics.

### Distinct clinical profiles of the two subtypes

**Fig. 4:**
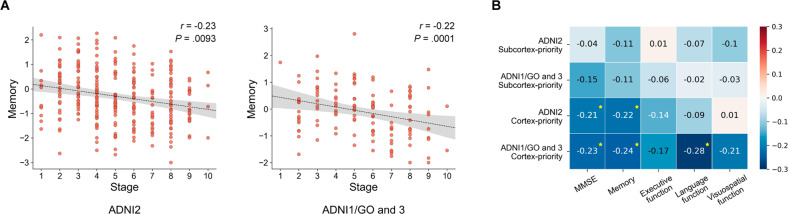
Subtypes exhibit distinct clinical profiles. **A** Progression stages of the cortex-priority subtype were associated with memory decline across the two datasets. **B** Characterization of the relationship between the progression stages of the subcortex-priority and cortex-priority subtypes and the clinical profiles. All associations were tested by Spearman correlation while controlling covariates (age, gender, and education). Values in white indicate *P* values below .05 and stars indicsate *P* values below 0.01.

Clinical profile comparisons between different subtypes across the discovery and validation datasets can be found in Table 2. Compared to the subcortex-priority subtype, individuals of the cortex-priority subtype showed worse memory, executive function, and language function. The significant differences, except for language impairment, were validated in the independent dataset. We also found that the stages of the cortex-priority subtype were highly correlated with memory decline across the two datasets (Fig. 4A). This association suggests that a higher stage is associated with worse memory. However, there was no significant and reproducible association between stages and these clinical profiles in the subcortex-priority subtype (Fig. 4B).

**Fig. 5:**
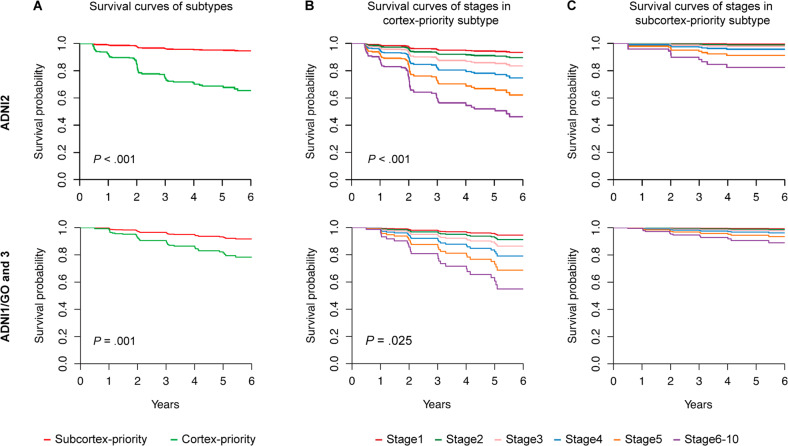
Subtypes and progression stages are associated with the risk of conversion to dementia. **A** Individuals with the cortex-priority subtype (red) had a higher probability of conversion to dementia compared to individuals with the subcortex-priority subtype (green) across the discovery and validation datasets. **B** High stages of the cortex-priority subtype identified very high-risk groups of conversion to dementia across the two datasets. **C** Survival curves comparing time-to-conversion of stages in subcortex-priority subtype across the two datasets. The later stages (6–10) are grouped into the last stage. All survival curve analyses were adjusted for the age, gender, and education covariates.

Next, we tested whether the two subtypes conferred differences in vulnerability to AD conversion. Survival curves of the subtypes are shown in Fig. 5. In the ADNI2 dataset, the individuals in the cortical-priority subtype had a higher risk of conversion to dementia than the subjects in the subcortical-priority subtype (hazard ratio: 7.62; 95% confidence interval: 4.03–14.39, *P* < 0.0001) within a mean follow-up period of 3.6 ± 1.84 years. This result was reproducible in the validation dataset (hazard ratio: 2.81; 95% confidence interval: 1.46–5.41, *P* = 0.0020) within a mean follow-up period of 3.9 ± 1.91 years. These findings suggest that the progression pattern of amyloid deposition in CN and MCI shows differences in cognitive decline. In the cortex-priority subtype, we found that individuals at higher stages had an increased probability of progressing to dementia compared to those at lower stages (hazard ratio: 1.63; 95% confidence interval: 1.31–2.01, *P* < 0.0001). Similar results were obtained in the independent dataset (hazard ratio: 1.59; 95% confidence interval: 1.06–2.41, *P* = .0255). By contrast, there was a lot of overlap between the conversion risk curves in the subcortex-priority subtype until the later stages (stage ≥ 6) in the cortex-priority subtype.

## Discussion

Here, we applied an unsupervised data-driven method in a broad range of CN and MCI individuals to evaluate the association between spatiotemporal variability in amyloid deposition and AD profiles. We identified two reproducible subtypes characterized by different regional progression patterns in two independent datasets. The spatiotemporal subtypes of amyloid deposition showed distinct cognitive and biomarker characteristics. Our findings suggest that spatiotemporal progression patterns in amyloid accumulation could provide insight into the disease monitoring and enrollment of therapeutic trials in CN and MCI.

In this study, the amyloid deposition was aptly described as two distinct progression patterns in a purely data-driven model, offering regional abnormality trajectories from early to late stages. In the cortex-priority subtype, the cingulate was the first region to show abnormality. In line with this, previous studies demonstrated that the cingulate has high-intensity values early on and may be the seed of amyloid deposition propagation [12, 26–28]. The following abnormal regions, the parietal lobe and frontal region, seem to be affected by the neighboring cingulate, and the association between them has been indicated in a previous study [29]. In general, the progression pattern of the cortex-priority subtype was similar to previous neuropathologic findings [8, 30, 31], except for the early appearance of the cingulate. In addition, some PET estimates showed that the medial frontal, medial parietal, and lateral temporo-parietal areas were the initial sites of amyloid deposition [32, 33]. Different ROI choices and analytic approaches may contribute to these inconsistencies. Data-driven methods may lead to unappreciated subtypes. The progression pattern in the subcortex-priority subtype was an intriguing result. In this subtype, the abnormalities in the subcortical regions occurred prior to those in the cerebral cortex. In particular, the thalamus and basal ganglia became abnormal earlier than the other regions. In line with this, previous studies have shown that the thalamus and basal ganglia are vulnerable to amyloid deposition and appear to have high SUVRs across preclinical AD phases [34, 35]. However, such a sequence contradicts the progression of amyloid from the neocortex to the subcortical regions in previous studies [14, 15, 36]. One possible explanation for the discrepancy is that the cutoffs we defined may make subcortical SUVRs less reliable and more sensitive to variation. Therefore, such subtype may fall short of explaining the ground truth neuropathology. Meanwhile, a distinct advantage of the present consideration is that it provided additional information of amyloid deposition and captured the early variation in the vast majority of individuals with few clinical symptoms.

The spatiotemporal subtypes enable linkage of amyloid accumulation trajectories with genetic and biomarker characteristics. *APOE* ε4 has consistently been found to be the strongest risk factor for AD, increasing the risk via oligomerization, aggregation, degradation, and clearance of amyloid [37]. Previous studies showed that individuals with positive amyloid-PET were more frequently *APOE ε4* carriers than those with negative ones [38]. Similar findings have been found in the two subtypes we identified. CSF indicators usually provide highly concordant information with PET measures [4, 39]. In line with this, we found significant associations between the stages and the CSF Aβ concentrations in distinct subtypes, but the CSF level was lower in the subcortex-priority subtype. Previous studies showed that amyloid-PET is associated with tau pathology in the preclinical and prodromal stages of AD [40, 41]. Because of the extensive absence of tau-PET data from the two datasets, we used CSF t-tau and p-tau data as measures of tau pathology and found that higher stages in either one of the subtypes were correlated with increased levels of t-tau and p-tau. Our findings indicate that all the sequences of regional vulnerabilities in amyloid deposition are associated with tau accumulation, potentially providing insight into the monitoring of AD neuropathology.

Few studies have examined the association between spatiotemporal variations in amyloid deposition and clinical presentation in CN and MCI. We found that individuals with the cortex-priority subtype had lower cognitive performance and executive function compared with those in the subcortex-priority subtype. Notably, individuals in the cortex-priority subtype had a higher probability of conversion to dementia within 6 years. Moreover, the stage may be a useful marker for cognitive decline or conversion risk for individuals in the cortex-priority subtype. However, there was no apparent conversion until the presence of cortical abnormality for individuals in the subcortex-priority subtype, providing evidence that a significant proportion of elderly subjects remain cognitively normal with amyloid deposition [42, 43]. Overall, participants with different progression patterns of amyloid accumulation have distinct disease trajectories in individuals in CN and MCI. Incorporating these findings into clinical trials with differentiated populations holds great promise for improving the accuracy of individualized diagnosis and providing opportunities for future therapeutic intervention to prevent or slow the rate of disease progression [44]. In particular, our findings suggested that amyloid abnormality in the cortical regions are a key predictor in the progression of cognitive decline and subsequent conversion to AD dementia.

Although we identified consistent spatiotemporal subtypes of amyloid burden across two independent datasets in CN and MCI that differed in disease profiles, our results need to be replicated in other cohorts. The subcortical regions are primarily being used to characterize the global amyloid accumulation. Current findings lack clear cutoffs of the subcortical area, especially in individuals with few clinical symptoms. Although the two subtypes identified by cutoffs defined in the context of our study resulted in more differentiated population stratification, future work is needed to balance the sensitivity and specificity to account for the underlying neuropathology. Furthermore, AD is a complex neurodegenerative disorder that is associated with amyloid deposition, tau accumulation, hypometabolism, brain atrophy, and a variety of biological processes. Future work should investigate other AD-related biomarkers to further improve risk stratification.

In summary, we found two spatiotemporal subtypes with regional progression patterns of amyloid accumulation in a broad range of individuals with few clinical symptoms. The amyloid subtypes showed distinct regional progression patterns and AD profiles. Furthermore, the regional progression patterns were associated with clinical and biomarker characteristics. Our findings highlight the importance of uncovering the spatiotemporal variations of amyloid deposition in CN and MCI for clinical trials and precision medicine.

## Supplementary information

Supplemental material

## Data Availability

The ADNI data used in this study were obtained from the ADNI database (available at https://adni.loni.usc.edu).
