# Supplementary material for: Distinct spatiotemporal subtypes of amyloid deposition are associated with diverging disease profiles in cognitively normal and mild cognitive impairment individuals
Source: Transl Psychiatry. 2023 Feb 2;13:35. doi: 10.1038/s41398-023-02328-2 (PMC9895066; doi:10.1038/s41398-023-02328-2)
Supplement: Supplementary file 1 — Supplemental material [file 41398_2023_2328_MOESM1_ESM.docx]

**List of Supplemental Materials**

1. **Supplementary Figure 1.**
2. **Supplementary Figure 2.**
3. **Supplementary Figure 3.**

**Supplementary Figure 1. Positional variance diagrams of progression patterns.**

**
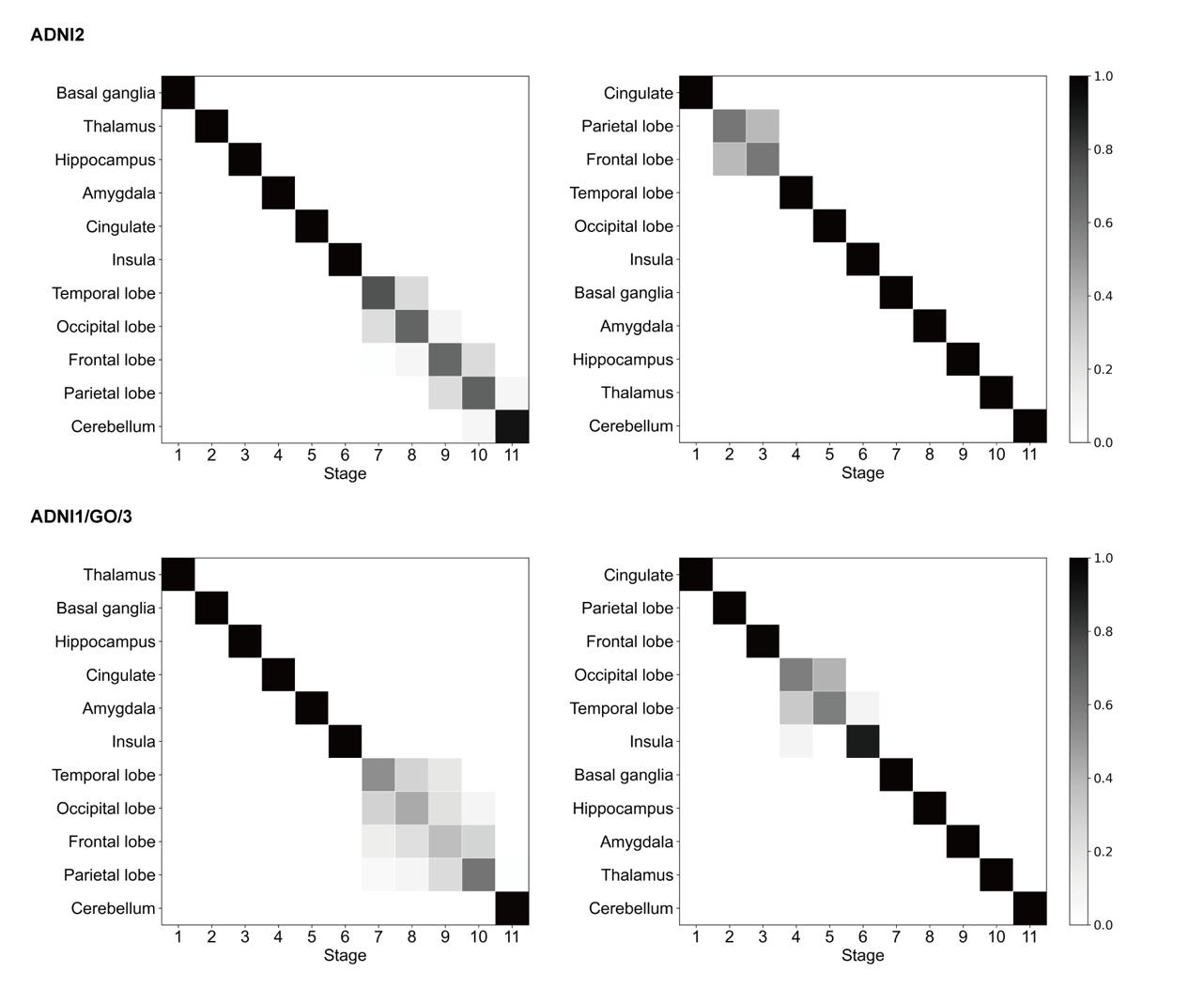
**

Sample candidate orderings from the optimal posterior distribution ordering by MCMC to evaluate the uncertainty of the trajectory of each subtype. Each row shows the frequency with which a region occupies each stage in the ordering over the set of samples from the MCMC. The uncertainty ranges from 0 in black to 1 in white.

**Supplementary Figure 2.** **Individual staging based on the subtypes.**


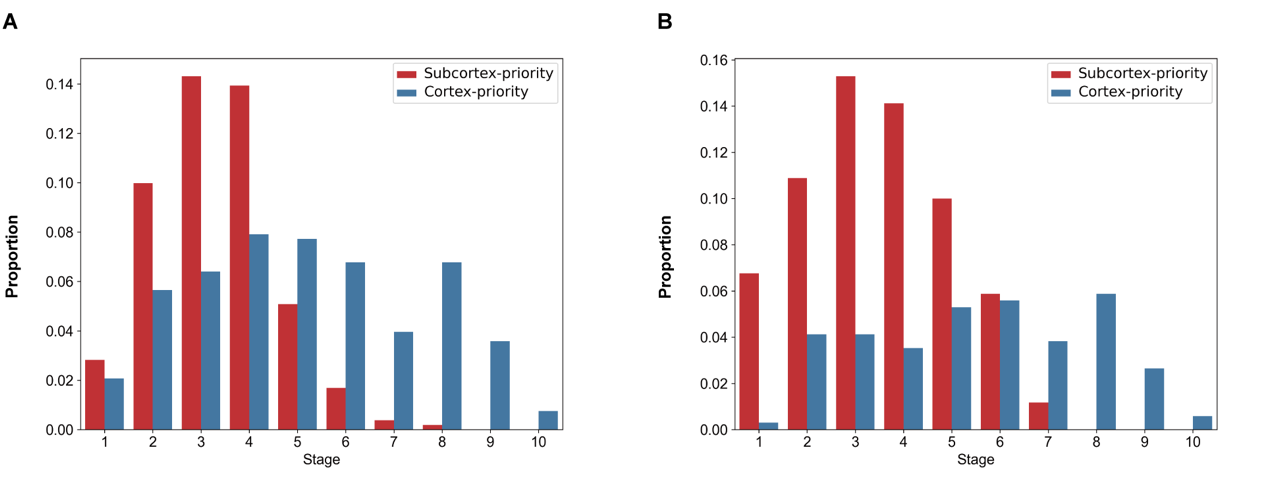


Distribution was acquired in ADNI2 (A) and ADNI1/Go and 3 (B). Staging individuals from CN and MCI in subcortex-priority subtype (red) and cortex-priority subtype (blue).

**Supplementary Figure 3. The distribution of CN and MCI in each dataset.**


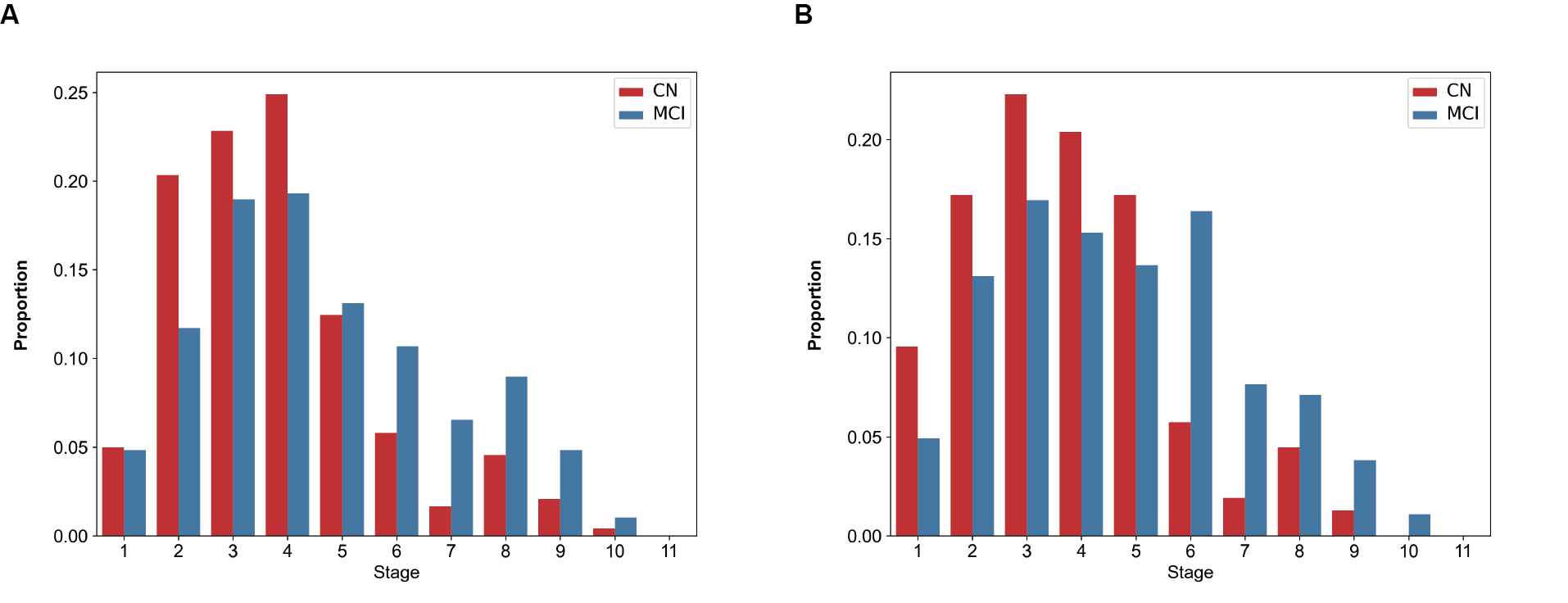


Distribution was acquired in ADNI2 (A) and ADNI1/Go and 3 (B).
